# A modular plug-and-play reaction platform for *operando* SEM investigations under fixed-bed conditions

**DOI:** 10.1039/d6cy00143b

**Published:** 2026-09-10

**Authors:** Luis Sandoval-Diaz, Johannes Zeininger, Daniel Delgado, Konstantinos Kappis, Frank Girgsdies, Maurits Vuijk, Annika Kubsch, Gudrun von der Waydbrink, Sebastian Matera, Günther Rupprechter, Karsten Reuter, Beatriz Roldan-Cuenya, Christoph Scheurer, Annette Trunschke, Thomas Lunkenbein

**Affiliations:** a Inorganic Chemistry Department, Fritz-Haber-Institute of the Max-Planck-Society Berlin Germany lunkenbein@fhi-berlin.mpg.de; b Chair of Operando-Analytics for Electrochemical Energy Storage, University of Bayreuth Bayreuth Germany Luis.Sandoval-Diaz@uni-bayreuth.de; c Institute of Materials Chemistry, TU Wien Vienna Austria; d Theory Department, Fritz-Haber-Institute of the Max-Planck-Society Berlin Germany; e Interface Science Department, Fritz-Haber-Institute of the Max-Planck-Society Berlin Germany

## Abstract

Fixed-bed reactors are widely employed in heterogeneous catalysis, providing controlled gas flow, efficient gas–solid contact, homogeneous heating and quantitative analysis of catalytic performance. Here, we present a modular platform for *operando* scanning electron microscopy (OSEM) inspired by these key design principles of fixed-bed reactors, while remaining compatible with the constraints of SEM operation. We demonstrate the system's capabilities using copper oxide reduction and the technologically relevant CO_2_ hydrogenation over a zincian malachite catalyst as benchmark reactions. We further explore how pressure influences product distribution during CO_2_ hydrogenation over a spinel catalyst. By combining the imaging ability of electron microscopy with quantitative performance analysis in the proposed configuration, the platform unlocks catalytic insights during activation, reaction and deactivation that were previously inaccessible to conventional SEM approaches.

## Abstract 2

The PPR assembly enables *operando* SEM of heterogeneous catalysts for direct correlation of structure with performance under reaction conditions.

## Graphical abstract

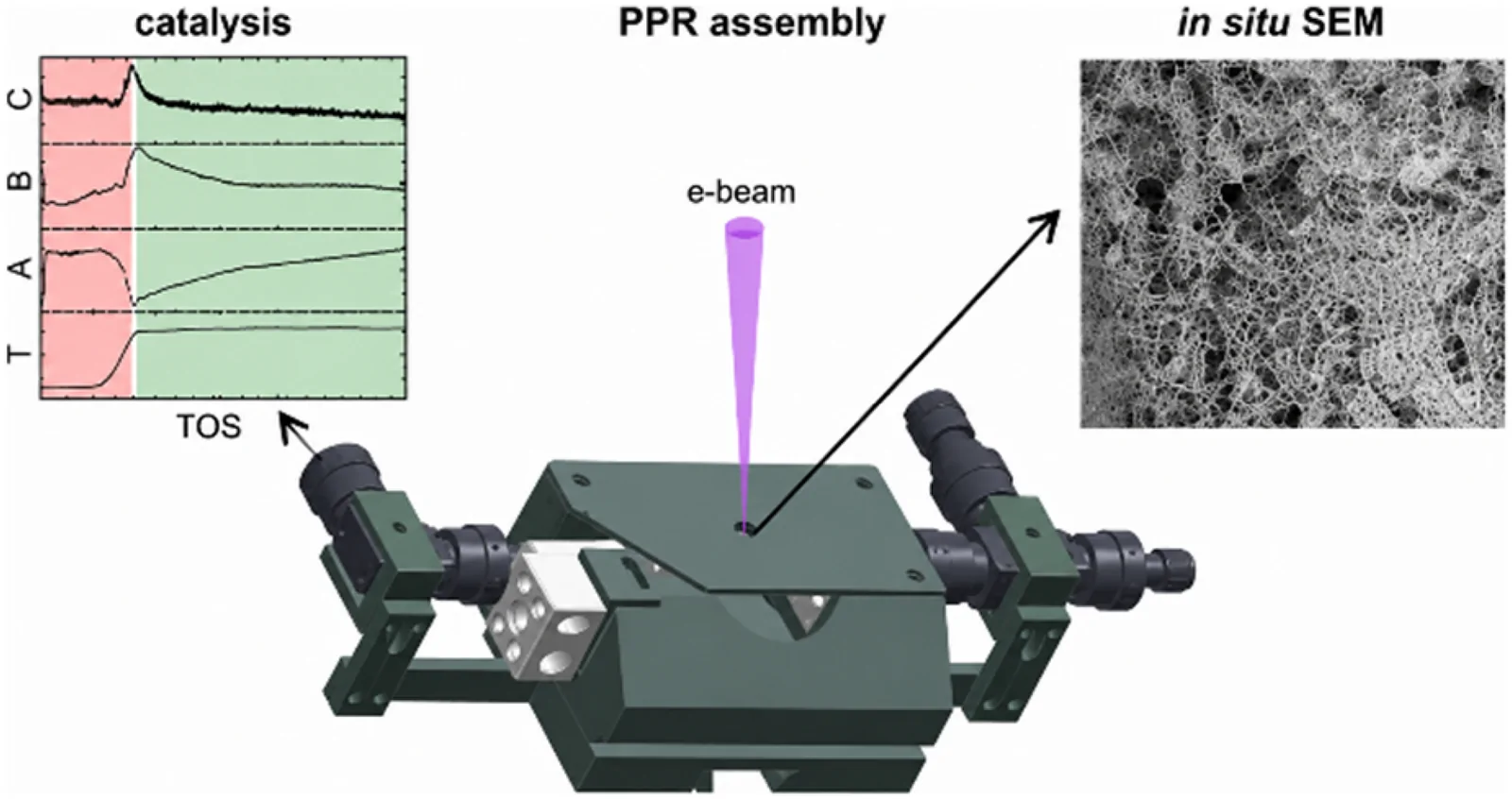

## 1. Introduction

*Operando* techniques are crucial for understanding catalytic phenomena.^1^ In this approach, the catalyst properties, such as chemical states and structures, are determined, while simultaneously the catalytic performance is monitored under reaction conditions.^2^ This extension to *in situ* methodologies allows to decipher relevant catalyst properties that correlate to the reaction behavior. In recent years, there have been significant advancements in this field, particularly after the realization that traditional vacuum-based surface characterization techniques can be combined with controlled gas environments inside the analysis chamber.^3^ This development enabled the *in situ* investigation of working heterogeneous catalysts under a variety of external conditions.

In this context, environmental scanning electron microscopy (ESEM) is a powerful tool for imaging localized surface structures of working catalytic materials.^4,5^ Like conventional SEM, ESEM uses an electron beam to probe the surface, but it can uniquely operate under relatively elevated pressures by employing differential pumping stages along the electron column. This configuration allows chamber pressures up to ∼40 mbar while maintaining adequate vacuum levels in the most sensitive parts of the instrument (∼10^−6^ mbar). However, imaging under the highest chamber pressure regimes of the ESEM presents several challenges. Beam scattering in the gas reduces image resolution,^4^ while signal transfer to the detector becomes highly sensitive to gas composition and pressure. In addition, the electron gun slowly saturates with gas after prolonged operation. These effects make it difficult to obtain stable, reproducible and high-quality images that can be directly correlated with the catalytic performance. Moreover, commercially available ESEM systems are not fully airtight, so that small amounts of atmospheric contaminants enter the chamber,^6^ altering the surface chemistry of sensitive catalysts.^7^ Consequently, while ESEM allows observation of catalysts under reactive gas environments, the achievable pressures, image stability, gas flow pattern and potential contamination limit its ability to provide reliable, quantitative structure–performance correlations, which are essential for *operando* studies.^1^

Here, we describe a customized reactor concept, the so-called plug-and-play reaction (PPR) assembly, that addresses the above-mentioned aspects for *operando* SEM (OSEM) studies in the ESEM. As we demonstrate, the PPR enables investigating catalyst materials under a fixed-bed reactor configuration, providing efficient gas–solid interactions and quantitative assessment of catalytic properties while simultaneously monitoring structural changes by SEM imaging. Furthermore, the PPR approach offers direct insight into the life cycle of catalysts, from activation to deactivation, under systematic variations of the reaction conditions, deepening our scientific understanding of catalytic phenomena.

## 2. PPR: the design concept

An essential component of any reactor is the excitation source, which enables, for instance, heating the system.^8^ For heating purposes in the (E)SEM, two approaches are commonly employed: laser-assisted SEM (LASEM) heating,^9–12^ and resistive heating. In LASEM, the sample is heated by laser illumination, enabling the generation of steep temperature gradients in both space and time. This heating mode is well suited for investigations of evaporation, melting, crystallization, precipitation, segregation, phase transitions, and cracking induced by thermal shock or thermal fatigue.^8,10^ On the other hand, resistive heaters may come in various forms, such as chips,^13^ microplates, semimetallic crucibles, and microfurnaces.^14^ Although resistive heaters are versatile and relatively cheap compared to LASEM, most designs feature a flat geometry. As a result, both LASEM and flat resistive heaters rely on unidirectional energy input over the sample, leading to significant temperature gradients. To mitigate this effect, the catalyst materials to be analyzed are typically deposited as thin, two-dimensional layers to maximize the thermal contact with the heat source. Under these conditions, only the surface directly exposed to the gas phase is catalytically active, leaving a substantial fraction of the catalyst underutilized, while a large portion of the gas stream bypasses the active surface. In contrast, catalytic beds comprise a three-dimensional distribution of particles through which the gas stream is forced to permeate. Hence, a larger number of active sites interacts with the reactant stream, while heat and mass transfer can be effectively controlled. Consequently, the fixed-bed configuration can enhance the catalytic conversion and improve the sensitivity for detecting small changes in catalyst performance.

**Fig. 1:**
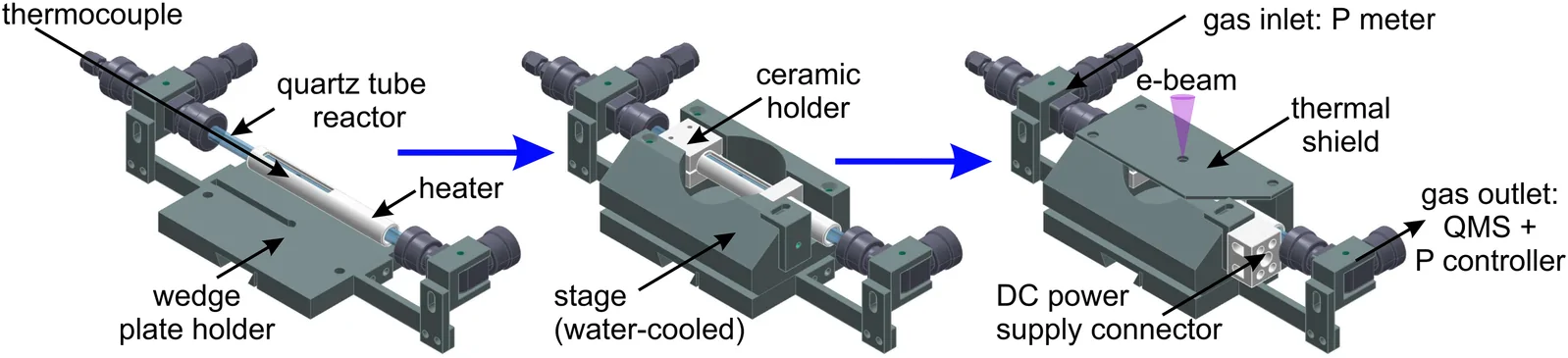
A representation of the PPR assembly for *operando* SEM investigations. The loaded quartz tube reactor is inserted into the customized tubular microheater. Due to thermal radiation, the PPR is housed in a water-cooled stage, and covered with a thermal shield preventing excessive emission of noisy thermal electrons.

Accordingly, our design consists of a fixed-bed reactor configuration integrated within the analytical chamber. As shown in Fig. 1, the PPR features a quartz tube reactor equipped with fittings for gas inlet and outlet as well as for temperature and pressure sensing. The (powdery) catalyst is loaded into the quartz tube, and a catalytic bed is formed by compaction with quartz wool or SiC powder, while ceramic spacers are used to adjust the position of the bed. The reactant stream is prepared in a dedicated gas-mixing station and introduced into the ESEM chamber *via* a feedthrough, from which it is fed into the PPR inlet. After interacting with the catalytic bed, the reaction gases exit the reactor and are directed into a divider fitting (“tee”) connected to a back-pressure controller and a quadrupole mass spectrometer (QMS).

The key of the PPR assembly is its customized resistive microheater made of a thin platinum path patterned onto an alumina foil. This foil is subsequently laminated and rolled into a tubular shape, as illustrated in Fig. S1, with an opening on the tubular wall for imaging purposes. Because the conductor is integrated within the alumina body, the metallic track is protected from the reactant gases while remaining electrically insulated both internally and externally. The temperature in the PPR is controlled by PID adjustment of a DC source connected to the heater terminals. For the OSEM application, the conductor path was manufactured with linear tracks running parallel to the heater axis (Fig. S1). In this geometry, the relative direction of the DC current applied to the heater terminals alternates with each adjacent path, hence canceling out the induced magnetic fields generated at the individual tracks. This conductor patterning is required to minimize the electromagnetic artifacts that may distort the electron imaging.

The PPR is housed in a water-cooled stage (Fig. 1) and secured onto a ceramic holder with screws. The assembly is covered with a metallic plate that serves as a thermal shield, into which a viewing port has been drilled. Furthermore, the heater is isolated from the stage with thermal ceramic wool. The reactor is ultimately mounted on a wedged plate compatible with our ESEM motorized stage. Images of the actual PPR assembly during a sample loading are presented in Fig. S2.

Since the electron beam cannot pass through the thick wall of the quartz tube reactor, a 1 mm circular hole has been drilled into this wall for SEM imaging. In this configuration, a portion of the gas flowing into the PPR leaks through the viewing hole into the microscope chamber, where it builds up the base pressure and serves as imaging medium. Because the gas stream flows from the reactor into the chamber, the active catalyst surface is protected from exposure to the chamber environment. The remaining gas that passes through the bed is detected at the reactor outlet by the QMS.

**Fig. 2:**
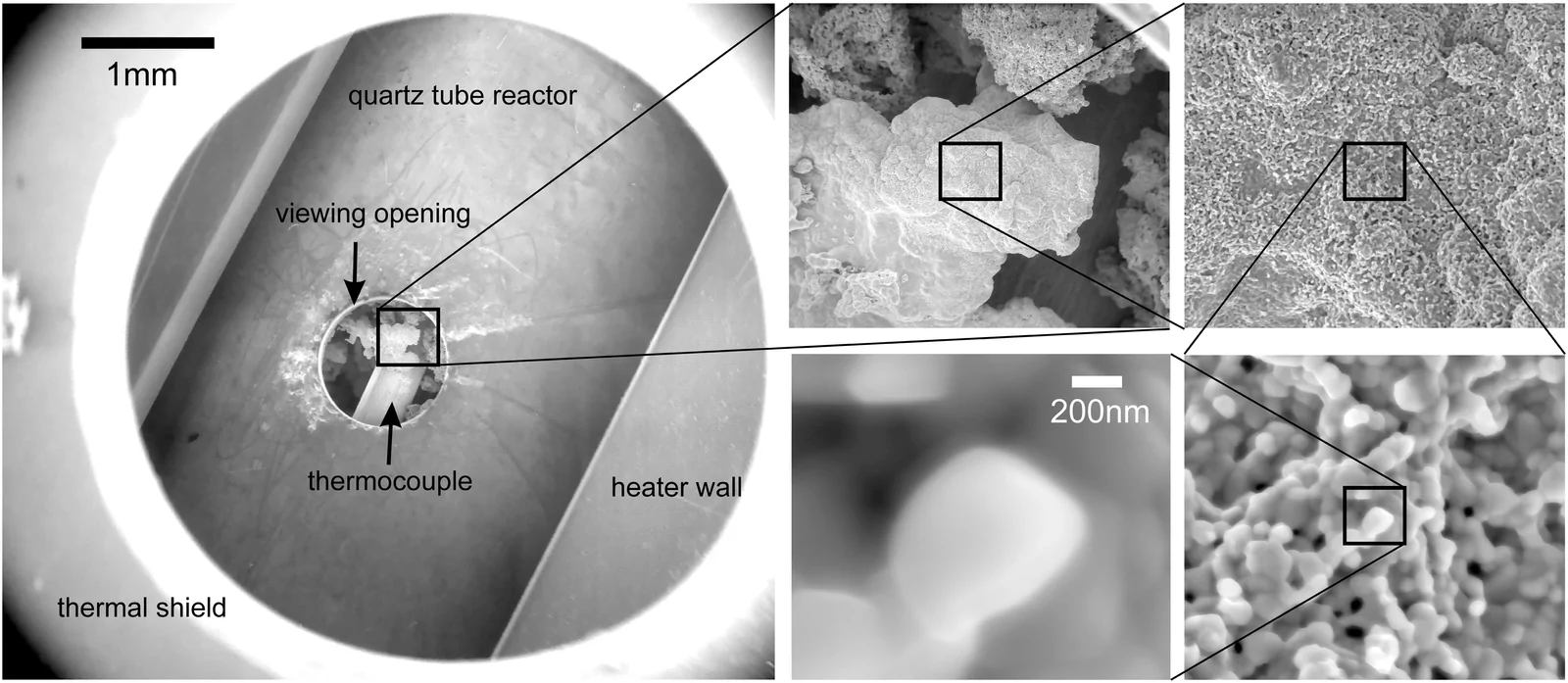
SEM imaging of a solid CuO sample loaded in the PPR assembly. The low resolution SEM image shows the different components of the assembly, including the viewing opening drilled into the quartz tube reactor wall, the heater wall, the thermocouple inserted in the bed, and the thermal shield. The insets display a series of progressively increasing magnifications revealing the CuO structures down to the nanoscale. Conditions of acquisition: RT, gas stream composed of 1 N_mL_ min^−1^ H_2_ : 4 N_mL_ min^−1^ Ar, chamber pressure 0.22 mbar, 10 kV.

Fig. 2 shows SEM images of the PPR assembly loaded with a powdery sample of CuO. The figure shows the thermal shield, the microheater wall, the quartz tube reactor, and the viewing port drilled onto the tube reactor wall providing an unobstructed view of the sample and the thermocouple inserted in the bed. Furthermore, the images at gradually increasing magnifications demonstrate the high-resolution SEM imaging of the CuO nanostructure.

## 3. PPR for *operando* SEM

We now discuss three examples illustrating the application of PPR in OSEM studies. First, we investigate the quantitative reduction of the CuO sample of Fig. 2. Next, we follow the life cycle of a zincian malachite catalyst during the technologically-relevant CO_2_ hydrogenation reaction. And finally, we investigate the reactivity of a Rh_0.1_MnFe_1.9_O_*x*_ spinel catalyst, also during the CO_2_ hydrogenation reaction, at varying temperatures and pressures.

### 3.1 Quantitative CuO reduction

**Fig. 3:**
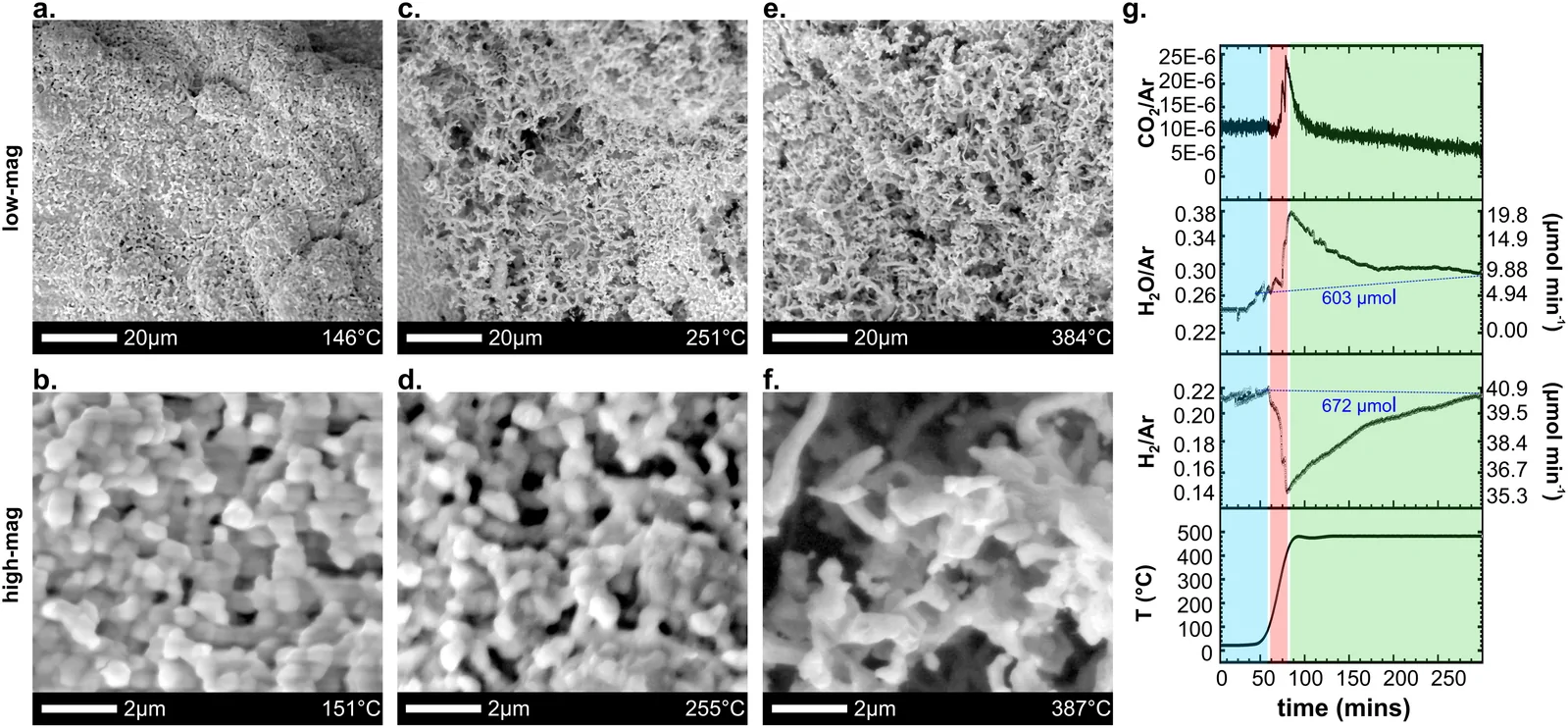
Transformation of solid CuO during ramping at 10 K min^−1^ in reductive gas stream investigated in the PPR. a. Low resolution SEM image of the CuO sample at 146 °C during temperature ramping. b. High resolution SEM image of the sample at 151 °C during temperature ramping revealing a polyhedral nanostructure. c. Low resolution SEM image of the sample at 251 °C during temperature ramping revealing dendrite-like morphologies. d. High resolution SEM image of the sample at 255 °C during temperature ramping revealing a transformation of the polyhedral nanostructure. e. Low resolution SEM image of the sample at 384 °C during temperature ramping revealing predominance of dendritic material. f. High resolution SEM image of the sample at 387 °C during temperature ramping revealing nanorod morphologies. g. Time series of the reaction traces displaying the PPR temperature and the ion currents detected in the QMS for H_2_, H_2_O and CO_2_ after normalization to Ar. Conditions of image acquisition: gas stream composed of 1 N_mL_ min^−1^ H_2_ : 4 N_mL_ min^−1^ Ar, chamber pressure 0.22 mbar, 10 kV.

In this example, we loaded the PPR with 54 mg of pure CuO. Morphologically, the micrometric material of this sample was found to be composed of irregular polyhedral nanoparticles of ∼200–500 nm fused together in a porous array (see Fig. 2). We dosed a gas stream containing 20% H_2_ in Ar and initiated the heating from room temperature (RT) to 484 °C under *in situ* SEM imaging. Afterwards, we kept the temperature at 484 °C for 200 minutes. As shown in Fig. 3a, the pristine catalyst structure observed at RT was still discernible after heating the sample to 146 °C in the reductive gas stream (compare to Fig. 2). A closer look, shown in Fig. 3b, shows the preservation of the characteristic polyhedral nanostructure of the oxide at this temperature.

With increasing temperatures in the range between 251 and 255 °C, the micrometer-sized particle observed in the overview image started to transform into a dendritic-like agglomerate (compare Fig. 3a and c), although some regions retaining the pristine compact morphology were still discernible. A closer view of this material is presented in Fig. 3d, revealing the reduction in size of the polyhedral nanoparticles accompanied by an increase in the open porosity. The polyhedral morphology of the nanoparticles started to be replaced by roundish shapes forming distinct branches of elongated agglomerates. At higher temperatures (384–387 °C, Fig. 3e), only the dendritic morphology was retained, giving rise to a foam-like structure at the micrometric scale, while the nanoparticles acquired the morphology of nanorods (Fig. 3f).

Due to the geometry of the PPR system, SEM imaging probes a region of the packed bed close to the reactor wall, where packing and associated local flow effects are expected to be most pronounced.^15^ Therefore, the local microscopic observations must be complemented by an evaluation of the overall behavior of the packed bed, for which integral gas-phase composition information is necessary. Accordingly, information about the reduction dynamics is presented in Fig. 3g, displaying the time series of the PPR temperature and the ionic currents of H_2_, H_2_O and CO_2_ (normalized to Ar) measured in the QMS. The plot shows that the signal of H_2_ decreased during the ramping, while the water signal increased, indicating the reduction of the sample (pink region of Fig. 3g). The H_2_ signal decreased up to 409 °C, and then increased slowly, and finally reached its initial level before the heating. This signal exhibited a marked tailing after the minimum, which implies a slow rate of reduction completion. We attribute this observation to the relatively low pressure of the experiment (∼0.22 mbar), involving a slow rate of reduction specifically of the bulk material. Hence, a long treatment of 200 minutes was required for completion.

Complementarily, the water production exhibited an opposite trace compared to H_2_. We calibrated and integrated the H_2_ and H_2_O signals by dosing known quantities of the gaseous probes to quantitatively assess the reaction progress. From these data, we determined an H_2_ consumption of 672 μmol and a water formation of 603 μmol. For comparison, the initial 54 mg of CuO corresponds to 676 μmol. This result indicates that the H_2_ signal provides a quantitative measure of the reaction progress, whereas the H_2_O signal reaches only 89% of the expected value, perhaps due to condensation losses in the reactor lines. Furthermore, it was possible to detect a very slight production of CO_2_ during the treatment, which could indicate trace carbonates or desorption of CO_2_ from the oxide material, in line with the expected high sensitivity of the PPR configuration.

We measured the temperature-programmed reduction (TPR) profile of this sample to get an idea of the reduction kinetics from an external data set (Fig. S3a and b). Although the exact line shapes differ between the PPR and the TPR experiments due to the different pressure regimes, the general trend in both measurements is similar. For instance, we detected that the reduction initiated at 153 °C in the PPR (Fig. 3g and S3c), which matches reasonably well with the determined value of 155 °C for the reduction onset obtained in the more conventional TPR experiment at 1 bar. We recovered the sample after the reductive treatment in the PPR and characterized it by powder X-ray diffraction (XRD, Fig. S4). The XRD patterns highlight the purity of the starting oxide and show that only metallic copper was present in the post-reaction material, corroborating the quantitative reduction achieved in the treatment. Overall, the correlative SEM and QMS data enable the local microscopic evolution of the material to be directly related to the integral reaction progress of the packed bed.

### 3.2 Monitoring the life of a zincian malachite catalyst during CO_2_ hydrogenation

In this example, we used a catalyst composed of Cu : Zn : Al of mole ratio 68 : 29 : 3.^16^ This material was obtained by coprecipitation of the metallic nitrates with Na_2_CO_3_ at a constant temperature (338 K).^17^ After spray-drying, the collected solid phase was found to correspond to a zincian malachite of homogeneous elemental distribution, low crystallinity, porous texture, and high specific surface area (∼120 m^2^ g^−1^). A comprehensive characterization of this catalyst has been published elsewhere.^16–18^

**Fig. 4:**
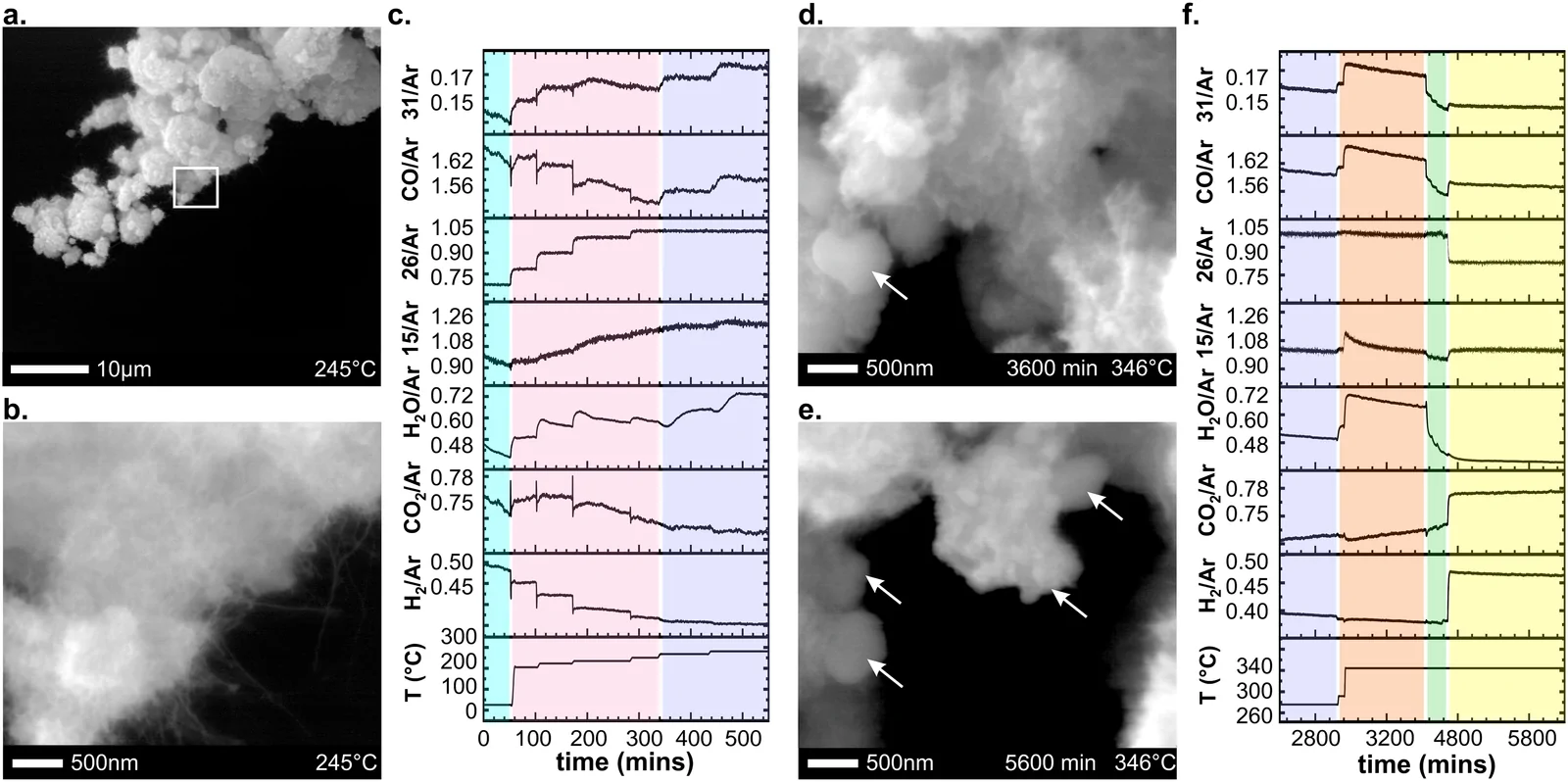
Transformation of zincian malachite catalyst during CO_2_ hydrogenation reaction. a. Low resolution SEM image of zincian malachite catalyst at 245 °C. b. High resolution SEM image of the region highlighted in a. revealing a cotton-ball-like structure formed by entangled nanofibers. c. Time series of the reaction traces displaying the PPR temperature (RT, 200 °C, 215 °C, 230 °C, 245 °C, 260 °C, 280 °C, ramping rate 10 K min^−1^) and the ion currents (normalized to Ar) detected in the QMS corresponding to H_2_, CO_2_, –CH_3_, H_2_O, –C_2_H_2_ and CO. d. High resolution SEM image at 346 °C showing the disintegration of the pristine nanofiber structure. e. High resolution SEM image at 346 °C showing further disintegration of the pristine nanofiber structure after 2000 minutes of treatment. The arrows show the agglomeration of the nanofibers in roundish micrometric particles. f. Continuated time series of the reaction traces presented in c. including the temperature steps to 300 °C and 346 °C. Conditions of image acquisition: gas stream composed of 6 N_mL_ min^−1^ H_2_ : 2 N_mL_ min^−1^ CO_2_ : 1 N_mL_ min^−1^ Ar, chamber pressure 0.35 mbar, 10 kV.

As shown in Fig. 4a and b, the catalyst is formed by nanofibers that are bundled together, giving rise to roundish particles at the micron scale. This cotton-ball-like structure was found to be preserved after heating stepwise from RT to 245 °C in a reaction mixture containing 6H_2_ : 2CO_2_ : 1Ar. During these treatments (Fig. 4c, pink region), the reaction traces of CO_2_ and H_2_ decreased after each temperature step, with concomitant production of hydrocarbons (*m*/*z* = 15 (–CH_3_), 26 (–C_2_H_2_)), methanol (*m*/*z* = 31), and water (see Table S1). In this temperature regime, CO production could not be observed clearly, probably due to the overlapping of its signal (*m*/*z* = 28) with the decreasing signal of CO_2_. Further temperature increments in the regime between 260 and 280 °C did not induce any appreciable changes in the catalyst morphology. Furthermore, the reaction traces of hydrocarbon fragments remained steady in this regime (Fig. 4c, purple region), while the signals of CO, methanol and water increased with the temperature steps. This observation indicates a variation in the reaction selectivity that could be described as a potentiation of reverse-water-gas-shift and methanol production at the expense of hydrocarbon formation (see Table S1). We kept the system for 2660 minutes at 280 °C, resulting in a steady and very slow deactivation of the reaction traces without any appreciable catalyst morphological evolution.

It is known that this type of catalysts may deactivate at temperatures above 300 °C.^19^ To understand this aspect, we increased the temperature to 300 °C for 40 minutes (experiment time = 3095 minutes), and finally to 346 °C (Fig. 4d–f). Notably, the catalyst nanostructure was found to be severely altered after an extended treatment at 346 °C, as shown in Fig. 4d. The images show the transformation of the nanofiber structure into highly compacted particles (see arrows in Fig. 4d) that became more compacted over time (Fig. 4e). A video showing this process is presented in the supporting information (Video S1). Accordingly, structural characterizations of this catalyst have shown the transformation of the initial zincian malachite into bulk Cu–Zn alloys following CO_2_ hydrogenation above 300 °C.^18^

Interestingly, the reaction traces in this high temperature regime exhibited a complex behavior (Fig. 4f). The temperature increments to 300 °C and then to 346 °C initially potentiated the formation of water, CO and methanol, but not the formation of hydrocarbon products. Furthermore, after the initial activation, the formation of water, CO and methanol started to decline steadily during the long treatment at 346 °C (Fig. 4f, orange region). This deactivation was initially slow and then became very fast after ∼1200 minutes at this temperature (green region of Fig. 4f). Ultimately, all the product fragment signals decreased, while the signals of the reactants, CO_2_ and H_2_, increased abruptly (yellow region of Fig. 4f). This observation indicates a dramatic loss of the catalytic activity following the catalyst morphological transformation into compacted particles.

It has been discussed that the active form of the Cu/ZnO catalyst involves some form of tuned metal–support interaction that stabilizes the electronic structure of the metallic component and imparts structural integrity to the active phase.^17,20,21^ With our results, it seems clear that excessively high temperatures induce the phase transformation of the nanofibers into compacted particles, probably due to Cu–Zn alloying,^18^ in which the metal–support interaction is no longer efficient. Hence, the observed decline of catalytic activity is a consequence of the catalyst's thermal instability in the chemical environment, inducing the loss of its active nanostructure.

### 3.3 PPR2: the effect of pressure on reaction kinetics

We have shown examples of the PPR operation with a viewing port of 1 mm diameter at pressure regimes below 1 mbar. To further understand this aspect, we measured the pressure at the inlet and at the outlet of the PPR for several gas flows, and found that the residual pressure was only marginally higher than in the ESEM chamber (see Table S2). To have an idea, we had to use very large flows (60 N_mL_ min^−1^) to reach pressure values of 0.92 mbar in the chamber, and 0.95 mbar at the PPR outlet. In addition, the SEM image quality usually deteriorated as the chamber pressure increased close to 1 mbar, which is a negative effect of working at increasing chamber pressures.

We approached numerically the data of these measurements (see second section of the SI) by combining Darcy's law with a representative-volume-averaged continuity formulation (see Fig. S6). We found that, with the 1 mm viewing hole, the pressures at the reactor inlet, at the viewing hole, and in the chamber were essentially identical, varying by less than approximately 2% for leaking gas velocities at the hole ranging from stationary conditions to above the speed of sound (Fig. S7). In brief, the 1 mm viewing hole was too large to efficiently confine the gas within the reactor, preventing the build-up of any pressure significantly higher than that of the microscope chamber.

To address this aspect, we prepared a second assembly, the PPR2, with a viewing port of only 100 μm diameter. Interestingly, the data in Table S2 show that a pressure of several tens mbar could be sustained in the PPR2 with only 9 N_mL_ min^−1^ of gas flow, while the pressure drop at the catalyst position remained between 85 to 75% of the inlet pressure depending on the target value at the pressure controller. At the upper bound, a gas flow of 30 N_mL_ min^−1^ resulted in a pressure of 120 mbar, which is the highest value controllable in our system. All in all, these results show that the smaller hole size enabled reaching much higher pressures compared to the initial PPR design.

**Fig. 5:**
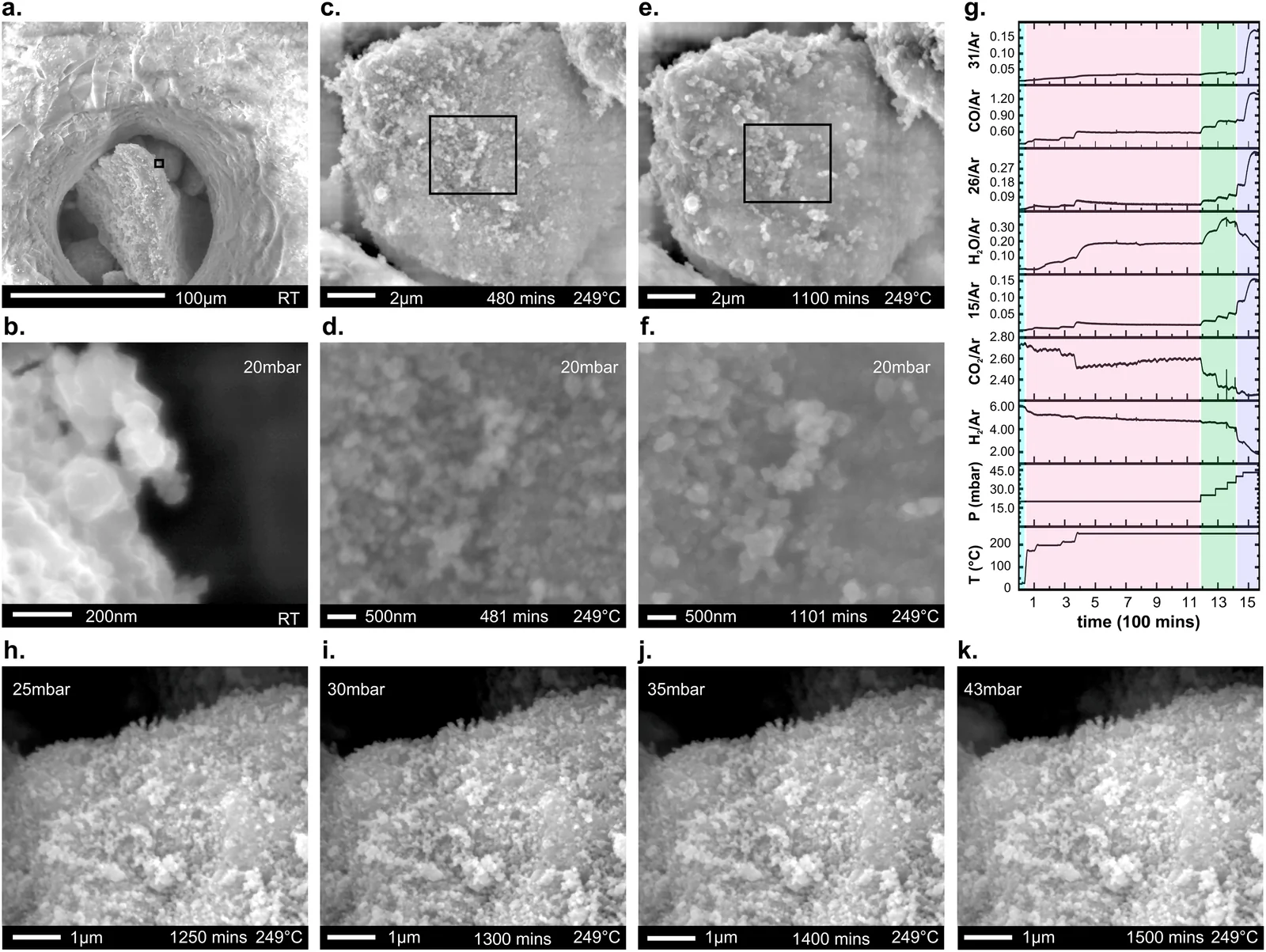
Transformation of a Rh_0.1_MnFe_1.9_O_*x*_ spinel catalyst and effect of pressure during CO_2_ hydrogenation reaction in the PPR2. a. A low resolution SEM image showing the 100 micron opening and the reactor wall of PPR2. b. A high resolution SEM image of the region highlighted in a. Showing the nanostructure of the spinel catalyst. c. A mid resolution SEM image showing a micrometric particle of the spinel catalyst at 249 °C. d. A high resolution SEM image of the region highlighted in c. Showing the nanostructure of the spinel catalyst. e. A mid resolution SEM image showing the micrometric particle of c. After 720 minutes at 249 °C. f. A high resolution SEM image of the region highlighted in e. Showing the nanostructure of the spinel catalyst after 720 minutes at 249 °C. a–f, PPR2 pressure = 20 mbar. g. Time series of the reaction traces displaying the PPR2 temperature (RT, 175 °C, 200 °C, 216 °C, 249 °C, ramping rate at 10 K min^−1^), pressure, and the ion currents (normalized to Ar) detected in the QMS corresponding to H_2_, CO_2_, –CH_3_, H_2_O, –C_2_H_2_, CO and –CH_3_O. h–k, SEM images of the catalyst under increasing pressures from 20 mbar to 43 mbar at 249 °C. Conditions of acquisition: gas stream composed of 6 N_mL_ min^−1^ H_2_ : 2 N_mL_ min^−1^ CO_2_ : 1 N_mL_ min^−1^ Ar, chamber pressure 0.16–0.18 mbar, 20 kV.

To test the concept under *operando* regime, the PPR2 was loaded with a MnFe_1.9_Rh_0.1_O_4_ catalyst designed for CO_2_ hydrogenation. An XRD analysis of the pristine material revealed a spinel structure, which partially transformed into a manganese carbonate phase after the reaction (Fig. S5). Notably, besides the narrow viewing port of the PPR2, Fig. 5a and the magnified region shown in Fig. 5b demonstrate good SEM imaging of the sample nanostructure. Furthermore, an internal pressure of 20 mbar could be sustained in the system, although the gas flows were the same as those used in the previous reaction example (total flow = 9 N_mL_ min^−1^). Hence, the operation pressure was effectively increased by two orders of magnitude with the PPR2. Importantly, the ESEM chamber pressure remained in the range between 0.16 and 0.18 mbar, which is a more beneficial regime for long-term imaging without the risk of electron column saturation or increased image noise.

We increased the temperature stepwise from RT to 249 °C in the reaction mixture without any pretreatment. The nanostructure of the pristine catalyst was found to be seemingly unaffected during these temperature increments, as shown in Fig. 5c and d. However, after ∼620 minutes at 249 °C, the nanostructure was altered (Fig. 5e and f). The changes at this stage can be described as an increase in nanoparticle sizes and a reduction in the number of nanoparticles (compare Fig. 5d and f). A video showing this transformation at a different catalyst region is presented in the supporting material (Video S2). This nanoparticle agglomeration after long exposure to the reaction conditions could point to the gradual transformation of the pristine spinel into the manganese carbonate type, as expected from the XRD characterization (Fig. S5).

Notably, the catalytic conversion also increased upon each temperature step, as shown in Fig. 5g (pink region). The plot shows that the pristine catalyst was already active to CO_2_ hydrogenation at 175 °C, as indicated by the reactants consumption and the increasing signals of hydrocarbon fragments, water, and CO. The conversion increased further during the successive temperature steps. In agreement with previous studies, these results show that Rh–Fe catalysts are primarily active for methanation and hydrocarbon formation during CO_2_ hydrogenation.^22^ Here, we corroborated this tendency, at least at temperatures between 175 and 216 °C. Nevertheless, a gradually increasing activity to methanol formation specifically above 216 °C was also observed. We think that the nanostructural transformation detected by the SEM imaging could be the reason of such catalyst activation to the methanol product. Afterwards, the system reached a steady level of catalytic activity that remained until the end of the treatment at 249 °C, although, as already mentioned, slow nanostructural changes took place during this period.

After ∼780 minutes of treatment at 249 °C (experiment time = 1190 minutes), we increased the reactor pressure stepwise to 25, 30 and 35 mbar, while keeping the same inlet gas compositions and reactor temperature. The data (green region of Fig. 5g) show that the conversion of CO_2_ and H_2_ increased upon each pressure increment, up to 35 mbar, potentiating both hydrocarbon and reverse-water-gas shift products, while the methanol signal remained steady. Subsequently, the reaction selectivities changed with further reactor pressure increments (purple region of Fig. 5g). At 40 mbar, the reactant conversion and the formation of hydrocarbons increased, while the water production started to decline. With the last pressure increment to 43 mbar, the water signal decreased even further, and a prominent signal of methanol was detected.

These observations indicate a definite change in the reaction selectivities with increasing reactor pressures, being 40 mbar the threshold for this transition. At the regime between 40 and 43 mbar, the evolution of water was hindered, while the methanol formation was significantly favored. This trade-off between water evolution and methanol synthesis can be understood from the reaction stoichiometries represented in Table S1. Water is generated in all of the listed reactions. However, hydrocarbon formation such as reactions 2–4 generate more water per hydrocarbon molecule compared to either the reverse water-gas-shift reaction or methanol synthesis. Hence, the potentiation of methanol production brings about intrinsically a reduction in water formation.

Obviously, the evolution of gaseous water becomes harder with increasing pressures. The water molecules that do not pass into the gas phase thus remain at the catalyst, favoring for instance the formation of an adlayer or hydroxylated functionalities at the surface, which may be key for methanol synthesis. Therefore, our results confirm the high sensitivity of methanol synthesis to the reaction pressure, which would have remained undetected by our analytics without the pressure control feature of PPR2. Notably, besides slight variations in the SEM image intensities, the image quality did not deteriorate following these pressure changes (Fig. 5h–k).

## 4. Concluding remarks and recommendations

In brief, we developed an ESEM-compatible microreactor that addresses many of the issues associated with *operando* SEM in heterogeneous catalysis. The fixed-bed inspired configuration of our PPR enhances the gas–solid interaction, thus providing a quantitative assessment of conversion while maintaining good SEM imaging resolution. The gas flow pattern further minimizes the influence of atmospheric contaminants normally found in the ESEM chamber, providing a controlled environment for the assessment of the catalytic properties. In addition, the selected pattern of the conductor path in the microheater minimizes electromagnetic image distortions, enabling high-resolution imaging during operation.

We disclosed the potential of the PPR with three examples of varying complexity. Starting with the reduction of a metallic oxide, we investigated its quantitative transformation into a metallic material. We went one step further with the investigation of the technologically-relevant CO_2_ hydrogenation reaction on a zincian malachite catalyst, correlating the deactivation of this material with the structural transformation of the active phase in the chemical environment. We furthered our study with an improved version of the PPR that enabled the generation of higher pressures around the catalyst, while maintaining the microscope chamber pressure at levels suitable for long-term imaging. Using again the CO_2_ hydrogenation as an example, we investigated the thermal activation of the reaction and observed a morphological transformation at the catalyst. We also investigated the effect of pressure on the reaction selectivities in the regime between 20 and 43 mbar, revealing a clear dependence of methanol formation on reactor pressure, accompanied by a reduced tendency toward water formation.

These results demonstrate the potential of the PPR approach for advancing *operando* SEM studies by extending the pressure range for reactive environments while maintaining direct image acquisition without the need for mathematical reconstruction or post-processing of raw images.^23^ Regarding the pressure distribution within the PPR2, the numerical modeling revealed that the pressure at the SEM imaging location remained at approximately 85 to 75% of the nominal value, providing an estimation of the local pressure conditions at the catalyst position and supporting the interpretation of *operando* catalytic measurements. The PPR2 can reach pressures of 120 mbar when using an inert gas mixture of 10 N_mL_ min^−1^ Ar : 20 N_mL_ min^−1^ N_2_, although under the reactive conditions investigated here, the pressure was limited to 43 mbar by the maximum gas flow rates available from our dosing system. We anticipate that future improvements will contribute to the closure of the pressure gap in heterogeneous catalysis.^1^

We acknowledge that electron irradiation during SEM imaging can induce local changes in the mechanical, electrical, and chemical properties of oxide materials even at relatively low electron doses.^24–26^ However, under OSEM conditions, charge dissipation through the gas atmosphere, continuous gas flow, and elevated temperatures distinguish the experimental environment from conventional high-vacuum SEM at room temperature. Therefore, although local beam-induced effects cannot be excluded, their extent under our *operando* conditions remains difficult to predict and should be addressed in future studies, for instance, by combining OSEM at varying electron doses with *in situ* spectroscopy.

Finally, we note that both PPR and PPR2 are compatible with backscattered electron (BSE) imaging in both vacuum and low-vacuum chamber modes. Although energy-dispersive X-ray spectroscopy (EDX) is, in principle, possible, the confined reactor geometry, particularly in PPR2, strongly limits the available X-ray collection solid angle due to shadowing by the reactor components. Achieving an adequate detector view would require tilting the entire assembly, increasing the risk of collision with the microscope components. Furthermore, the EDX detector of our instrument cannot be operated above approximately 50 °C. Consequently, *in situ* EDX measurements were not performed with the PPR configurations, and their implementation requires further development beyond the scope of the present study. Complementary *ex situ* EDX and *in situ*/*operando* near-ambient-pressure X-ray photoelectron spectroscopy (NAP-XPS) measurements are currently preferred for obtaining associated chemical information.

## 5. Methods

### 5.1 PPR manufacture

The customized heater cartridges of the PPR assembly were acquired from Rauschert Gruppe, with 7 mm ID and 100 mm length, and a hot region of 55 mm. The resistance of the heater ranged between 72 and 75 ohm at room temperature. The rest of the assembly was manufactured internally at the Fritz-Haber-Institute's workshop. The PPR2 was manufactured by thinning the quartz tube (OD = 6 mm, ID = 4 mm) wall with a diamond saw, and then the 100 μm opening was carved by focused laser ablation. For this, the tube was placed into a LIBS J200 system (Applied Spectra Inc., USA) equipped with a frequency-quadrupled Nd:YAG laser (Quantel Ultra, Quantel, USA) emitting at 266 nm with a pulse width of 8 ns. The hole was created by ablating the tube in air with a spot size of 100 μm, with a repetition rate of 20 Hz, and a pulse energy of 7 mJ for a duration of 10 s. Afterwards, the opening was smoothed out with a 100 μm diamond drill. For the water-cooled stage, we used a commercial ESEM hot stage from FEI, from which we removed its previously damaged heater, and refilled this cavity with thermal isolating wool and the microheater assembly.

### 5.2 *Operando* SEM setup

For the *operando* SEM imaging, we used an FEI Quanta 200 FEG, with dedicated flanges for gas, water and electricity feedthroughs. The outlet of the PPR assembly was lined to a dividing tee, leading in one end to a quadrupole mass spectrometer (QMS, Prisma 200 Pfeiffer) and in the other end to a back pressure controller (Bronkhorst,1–120 mbar). Pure gases (Westfalen 5.0) were dosed into the quartz tube reactor by individual mass-flow controllers (Bronkhorst), and the gas lines except for CO_2_ were equipped with O_2_/moisture filters (Agilent in-line purifier OT3-2) to ensure gas quality. A pressure meter (Bronkhorst) was installed at the gas feeding line before the PPR inlet. The catalyst temperature was measured with a K-type thermocouple inserted in the catalytic bed. The temperature of the reactor was controlled by a process controller unit (Eurotherm EPC3016) by adjusting the power of a DC source (RS 771-7724, MeanWell LCM-60) lined to the heater terminals.

After loading the catalyst, the PPR was purged for several hours until the QMS signals associated with residual atmospheric species (*e.g.*, H_2_O and O_2_) reached stable baseline values, ensuring reproducible initial conditions prior to catalytic measurements. For all temperature-programmed experiments, the temperature was increased stepwise at a rate of 10 K min^−1^, unless otherwise stated.

Images of the catalyst surface were acquired with the large field detector of the ESEM every 17–68 s at acceleration voltages of 10–20 kV with a pixel depth of 8 bits. During the experiments, potential beam-induced changes in the gas phase composition were routinely assessed by switching the electron beam on and off while simultaneously monitoring the QMS signals. Under the investigated conditions, no measurable changes in the gas phase composition could be correlated to electron-beam exposure. The collected video frames were treated with Fiji.^27^ The stack of video frames was aligned with translation registration. Afterwards, the image noise was reduced with a bilateral filter (min: 4, max: 56).

### 5.3 Sample preparation

The solid CuO sample (99.9%) was acquired from Sigma-Aldrich and used without any further treatment.

The zincian malachite sample was synthetized in the study referenced in ref. 16 and directly used in the experiments.

The MnFe_1.9_Rh_0.1_O_4_ spinel was synthesized by hydrothermal decomposition of oxalates, using Mn(ii) nitrate tetrahydrate (Sigma-Aldrich, >97%), iron(iii) chloride anhydrous (Sigma-Aldrich), Rh chloride hydrate (Sigma Aldrich, 98%), oxalic acid dihydrate (Sigma-Aldrich, 99%), sodium hydroxide (Sigma-Aldrich, 99%) and tetrabutylammonium hydroxide (AppliChem, 20% w/w in H_2_O). Aqueous solutions (132.6 mL of deionized water) containing stoichiometric amounts of the corresponding metal salts and oxalic acid (Mn^2+^ : (Fe^3+^+Rh^3+^) : oxalic acid, 11.75 : 23.50 : 35.25 mmol) were alkalinized with 10 M NaOH (final pH = 10.3). Subsequently, 23 mL of the tetrabutylammonium hydroxide mixture was added and the obtained suspensions were introduced into an autoclave HPM-PT-04 (400 mL) made of Hastelloy C22 (Premex Reactor GmbH) and treated at 140 °C for 12 h under autogenous pressure at a heating rate of 3 K min^−1^, a cooling rate of 1 K min^−1^, and a stirring rate of 100 rpm. The resulting suspension was centrifuged, and the solid fraction was recovered and washed, and centrifuged again, 6 consecutive times with *ca.* 240 mL of H_2_O. The resulting solid was dried at 80 °C for 20 h, and subsequently heat-treated under Ar flow (100 mL min^−1^) at 500 °C for 2 h in a tubular furnace (heating rate 3 K min^−1^).

## Author contributions

LSD and GvdW designed the PPR. LSD, JZ, MV and AK performed the OSEM experiments. DD and KK synthetized and analyzed the spinel catalyst. DD and KK performed the TPR measurements. LSD, DD and FG performed and analyzed the XRD measurements. SM performed the numerical modeling of the reactor pessure profile. AT, CS, BRC, KR, GR and TL guided the research and commented on the final manuscript version. LSD wrote the manuscript.

## Conflicts of interest

The authors declare no conflicts of interest.

## Supplementary Material

CY-016-D6CY00143B-s001

CY-016-D6CY00143B-s002

CY-016-D6CY00143B-s003

## Data Availability

Data for this article, including QMS data and ESEM frames, are available at the AC/CatLab Archive at https://ac.archive.fhi.mpg.de/P52184. Supplementary information (SI) is available containing additional details about the PPR assembly and operation, catalyst characterization, numerical modelling of pressure gradients in the reactor, and residence-time estimations. See DOI: https://doi.org/10.1039/d6cy00143b.
