# Supplementary material for: A modular plug-and-play reaction platform for operando SEM investigations under fixed-bed conditions
Source: Catal Sci Technol. 2026 Sep 10. Online ahead of print. doi: 10.1039/d6cy00143b (PMC13580394; doi:10.1039/d6cy00143b)
Supplement: Supplementary file 1 [file CY-OLF-D6CY00143B-s001.pdf]

This journal is © The Royal Society of Chemistry 2026

Supporting information to

A Modular Plug-and-Play Reaction Platform for Operando SEM Investigations under Fixed-Bed Conditions

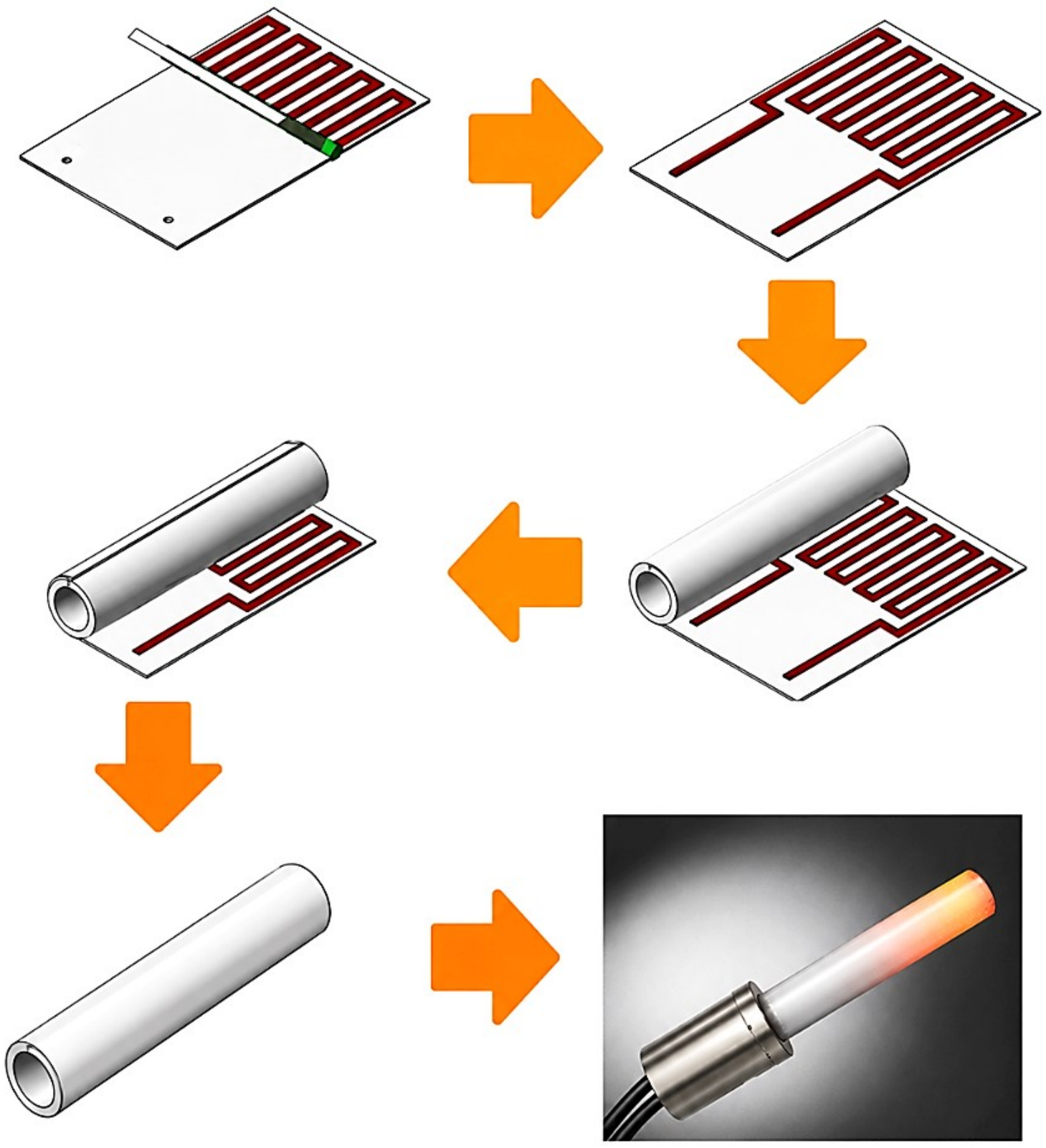

Fig. S1. An overview of the customized heater manufacture. The figure highlights the printing of the conductor path, the rolling, and the operative device (10 cm long for PPR application). Taken from Rauschert Gruppe.

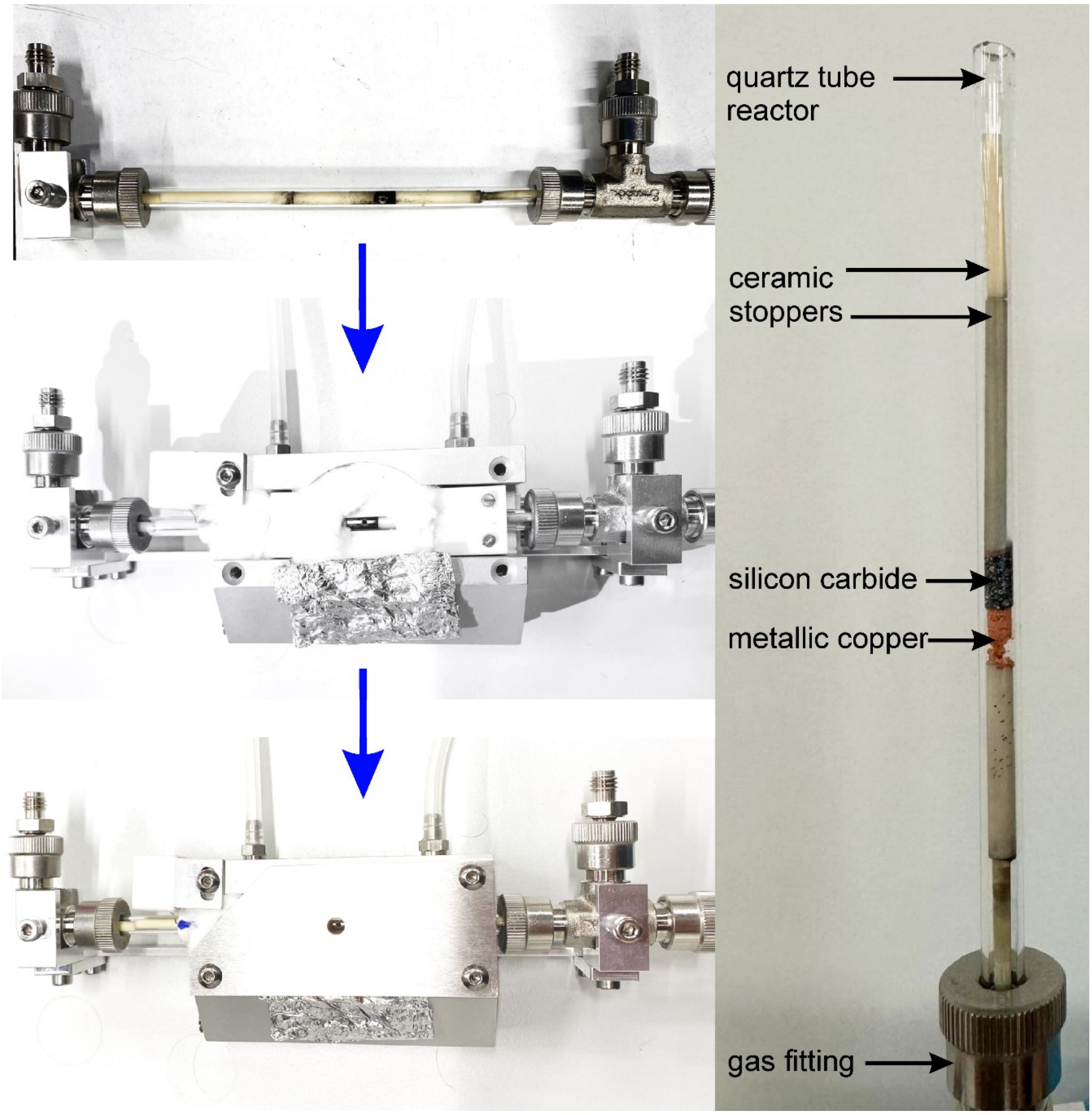

Fig. S2. Mounting and loading of the actual PPR assembly. The figure shows the loaded catalytic bed, the ceramic spacers, the cooling stage housing and the installation of the heat shield. The image on the right shows typical fixed bed material after PPR loading.

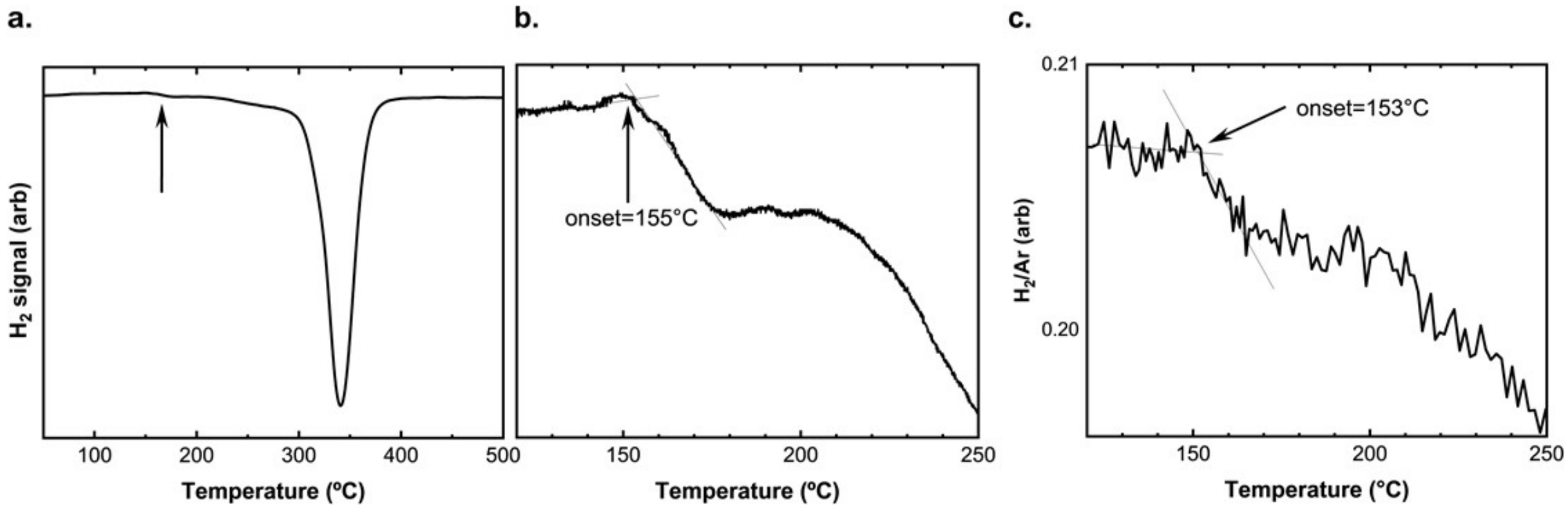

Fig. S3. A comparison between the reduction of a CuO sample in a conventional TPR reactor and in the PPR. a. TPR profile of 51mg of CuO sample at $5 Kmin^{-1}$, $5\% H_2$ diluted in Ar, total flow= $80 N_{mL} min^{-1}$, and pressure= 1 bar. The arrow in **a.** marks the beginning of the reduction. **b.** A magnified portion of the dataset presented in **a.**, showing the initiation of the reduction at 155 °C. **c.** The H_2 signal (normalized to Ar) detected in the PPR shows a reduction onset at 153 °C and a very similar profile compared to **b**.

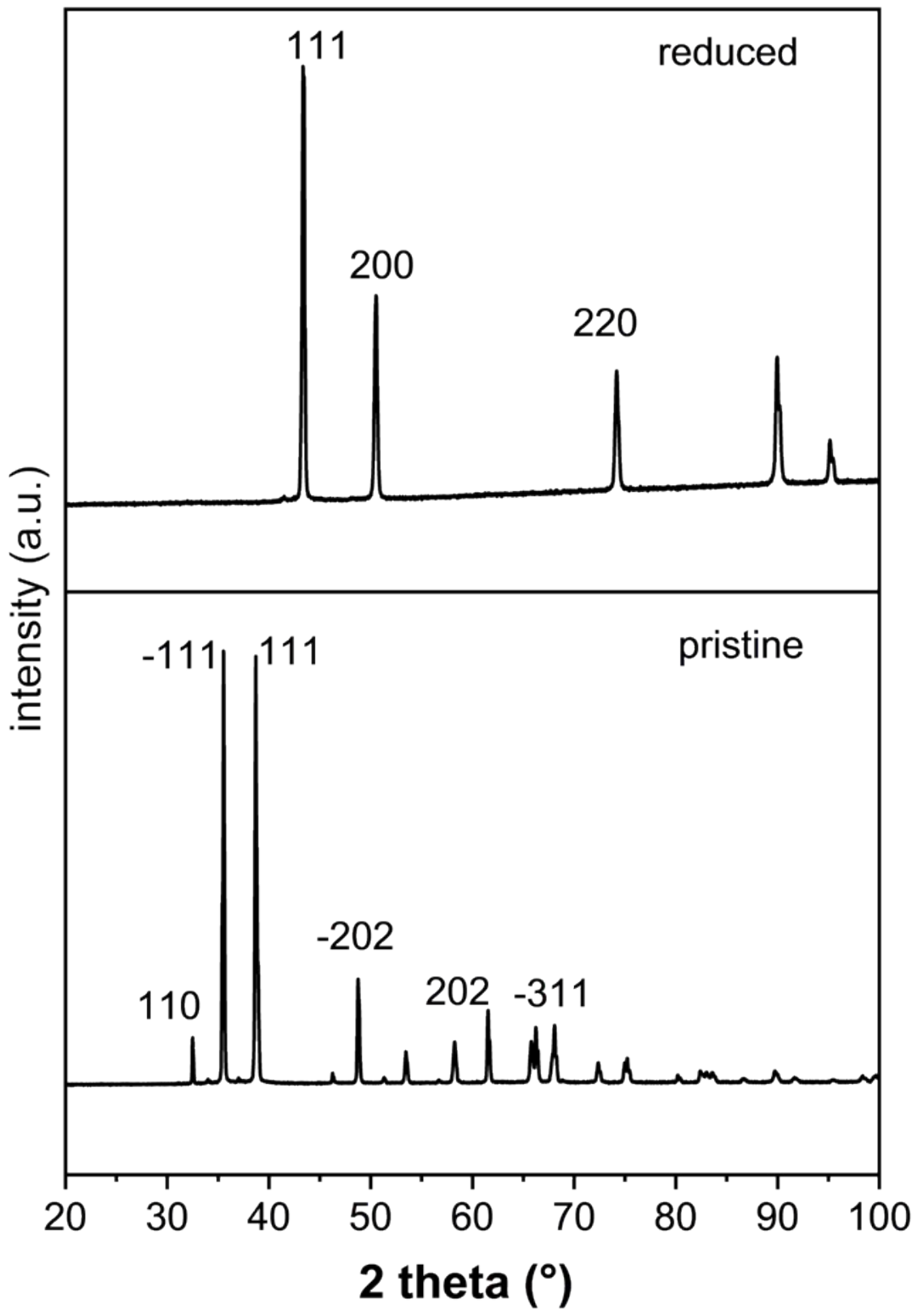

Fig. S4. Powder XRD patterns of the copper oxide material prior and after the reductive treatment in the PPR. The patterns show the quantitative transformation of CuO into metallic Cu.

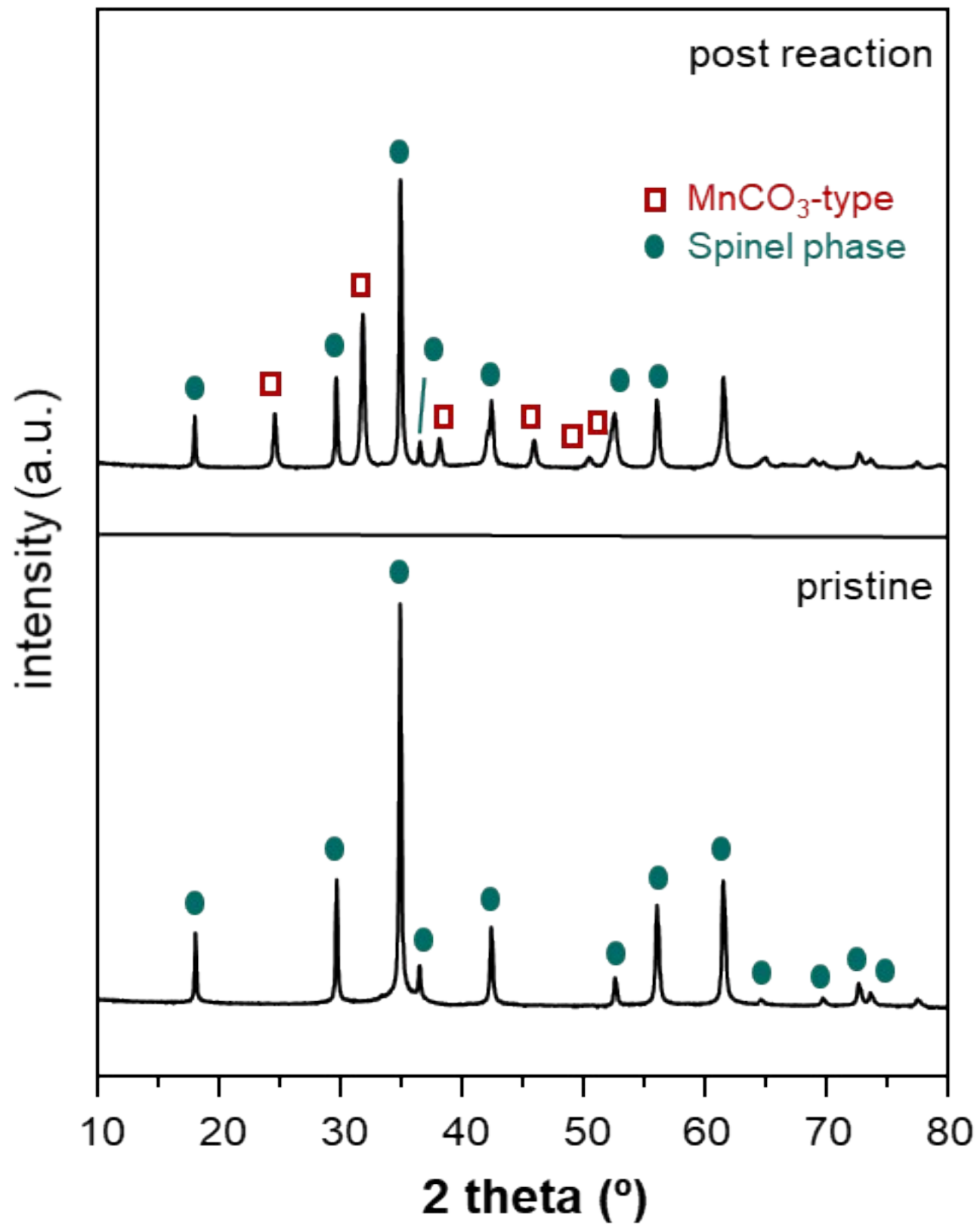

Fig. S5. Powder XRD patterns of pristine and post-reaction $MnFe_{1.9}$ $Rh_{0.1}O_4$ spinel catalyst. The patterns reveal that the spinel crystalline phase partially transformed into a manganese carbonate-type following catalytic hydrogenation of CO_2.

Tab. S1. A list of the reactions representing different stoichiometries of CO_2 hydrogenation and their correlation to QMS fragments.

**Reaction number**	**Reaction name**	**Reactants**	**Products**	**Product fragment (m/Z)**
**1**	**RWGS**	$CO_2 + H_2$	**CO** + H_2O	**28** + 18
**2**	**Sabatier methanation**	$CO_2 + 4H_2$	$\mathbf{CH_4}$ + $2H_2O$	**15** + 18
**3**	**Acetylene formation**	$2CO_2 + 5H_2$	$\mathbf{C_2H_2}$ + $4H_2O$	**26** + 18
**4**	**Ethylene formation**	$2CO_2 + 6H_2$	$\mathbf{C_2H_4}$ + $4H_2O$	**26** + 18
**5**	**Methanol formation**	$CO_2 + 3H_2$	$\mathbf{CH_3OH}$ + H_2O	**31** + 18

Modelling Pressure Gradients in the Catalyst Bed

A schematic representation of the reaction chamber is shown in Figure S1. The reactor consists of a quartz tube with an inner diameter of 4 mm. A viewing hole is drilled at the center of the tube to allow SEM imaging while simultaneously providing a controlled gas leak into the surrounding SEM chamber, which is maintained under near-vacuum conditions. Depending on the experimental configuration, the hole diameter is either 1 mm (PPR1) or 0.1 mm (PPR2).

The catalytic bed occupies a length of 6 mm and is positioned directly beneath the viewing hole. Gas enters the bed from the inlet with a prescribed mass flux f and pressure p_{in}, defined at the interface between the free-flow region and the porous catalyst bed. The gas exits both through the reactor outlet and through the viewing hole. The objective of the model is to estimate the pressure at the observation window, p_{hole}, using the experimentally measured inlet and outlet pressures together with the gas flow conditions.

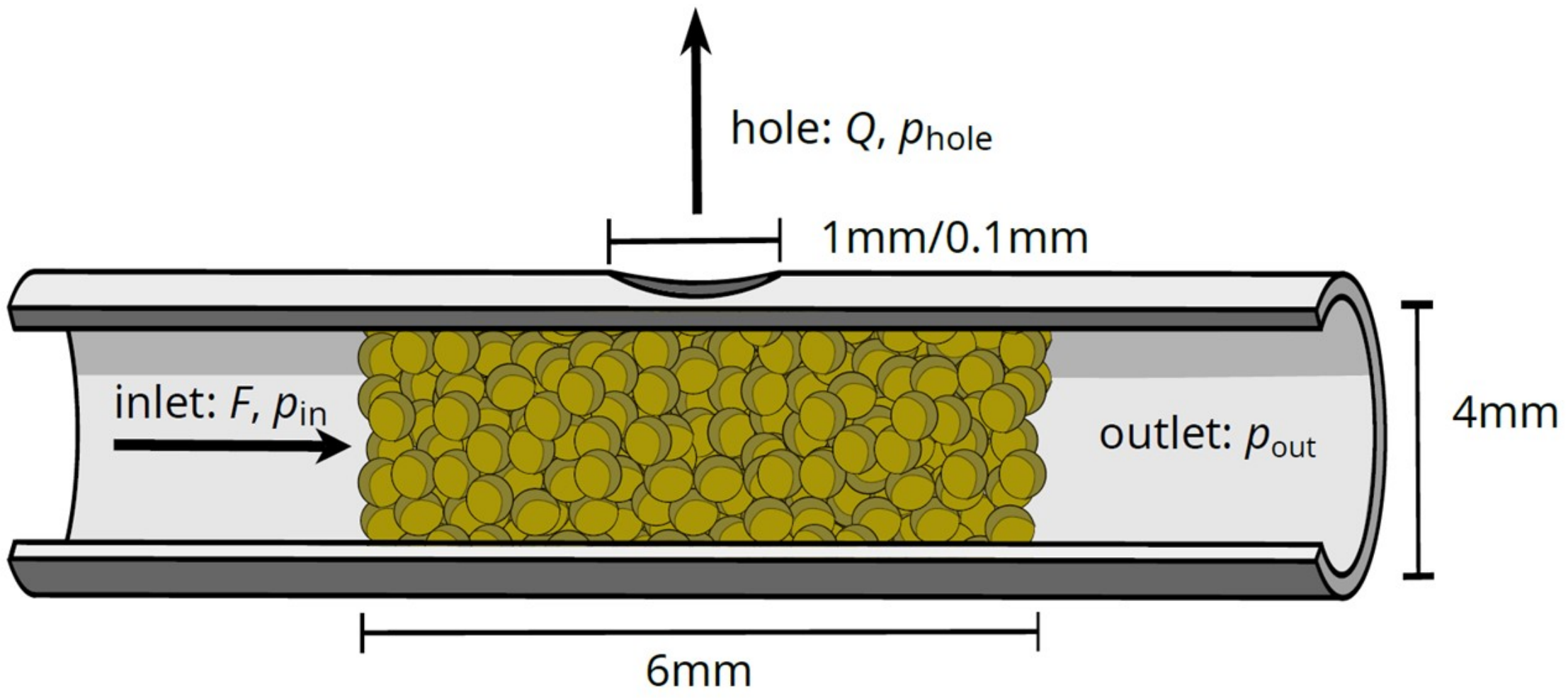

Fig. S6. A representation of the PPR longitudinal cross section representing the catalyst packing and the most relevant parameters used in the numerical modeling.

To describe gas transport within the catalyst bed, the porous medium is modeled as an isothermal, stationary porous material using a representative-volume-averaged continuum formulation [1]. In this approach, each representative volume element contains a sufficiently large number of catalyst particles such that density variations within the element can be neglected.

Gas transport is assumed to obey Darcy's law, which states that the gas velocity is proportional to the pressure gradient within the porous medium. Combining Darcy's law with the continuity equation and the ideal gas law yields the following governing equation for the pore pressure:

$$\nabla \cdot \left(p \frac{\eta k}{\mu} \nabla p \right) = 0 \qquad (1)$$

where:

- p is the gas pressure,
- η is the bed porosity,
- k is the permeability,
- μ is the gas viscosity.

The catalyst bed occupies the domain Ω, bounded by the inlet, outlet, impermeable reactor wall, and the observation hole. The reactor wall is assumed to be impermeable, implying zero normal mass flux. At the inlet and observation window, constant mass fluxes are prescribed, while the outlet pressure is fixed to the experimentally measured value.

To simplify the mathematical formulation, we exploit that gas viscosity is essentially independent of pressure under the investigated operating conditions [2-4]. Likewise, both porosity and permeability are assumed constant throughout the catalyst bed.

Introducing the auxiliary variable

$$u = \frac{\eta k}{2\mu} p^2 \qquad (2)$$

the nonlinear pressure equation can be transformed into the linear Laplace equation

$$\Delta u = 0 \qquad (3)$$

This transformation considerably simplifies the numerical solution of the problem.

The solution can then be expressed as

$$u = u_{out} + F u_F - Q u_Q \qquad (4)$$

where u_F and u_Q are solutions of two elementary boundary-value problems corresponding to unit inlet flux and unit outlet leakage through the observation hole, respectively.

An important advantage of this formulation is that, for a given reactor geometry, only these two linear problems need to be solved numerically. For any given geometry, we thus have to solve just two linear boundary value problems irrespective of the values of the parameters F, Q and $\eta k/\mu$. Solutions for arbitrary operating conditions are subsequently obtained through linear superposition.

Because the theoretical model is intended as a first-order approximation, the cylindrical reactor geometry was simplified. The circular tube was replaced by a square channel having the same cross-sectional area (approximately 3.5 mm × 3.5 mm). Likewise, the circular observation hole was represented by a slit extending across the reactor width, with an area equal to that of the original circular opening.

Using a coordinate system in which the x-axis is aligned with the gas flow direction and the y-axis is normal to the observation slit, the problem becomes two-dimensional, allowing efficient numerical solution.

The function corresponding to the inlet contribution can be obtained analytically as

$$u_F = -(x - L/2) \qquad (5)$$

where the coordinate origin is located at the center of the observation slit.

The second basis function, u_Q, was solved numerically using the adaptive finite-element software KARDOS [5, 6], employing piecewise linear basis functions and adaptive mesh refinement until an estimated numerical error below 10^{-11} was achieved.

With the solutions for u_F and u_Q, we can solve for the pressure at the hole

$$p_{\text{hole}}^2 = p_{\text{out}}^2 + \frac{0.5F\, u_{F,\text{in}} + Q\, u_{Q,\text{hole}}}{F\, u_{F,\text{in}} + Q\, u_{Q,\text{in}}} \times \left(p_{\text{in}}^2 - p_{\text{out}}^2\right) \quad (6)$$

where F can be determined from the temperature and the pressure at the inlet p_{in}. What remains are senseful values for Q. For this we exploit that

$$Q = p_{\text{hole}}\, v_{\text{hole}} \quad (7)$$

Experimental Conditions Used in the Simulations

Tab. S2. A list of experimentally determined pressure values at several flow rate and temperature conditions for PPR and PPR2.

**Reactor**	**Case**	**Hole diameter (mm)**	**Temperature (K)**	**Flow rate (Nml min^{-1})**	**Inlet pressure (mbar)**	**Outlet pressure (mbar)**	**Chamber pressure (mbar)**	**Estimated residence time (ms)$^{\$}$**
PPR	1	1	763	5	0.22	0.22	0.22	--
	2	1	619	9	0.36	0.35	0.35	--
	3	1	293	60	0.96	0.95	0.92	--
PPR2	4	0.1	522	9	21	20	0.16	2.1
	5	0.1	522	9	41	40	0.18	4.1
	6	0.1	522	9	45	43	0.18	4.9
	7	0.1	298	30	125	120*	0.37	3.8

* Upper value with used back pressure controller model. $ Estimated for 40% void volume and ideal gas behavior.

The simulations were performed using the operating conditions listed in Tab S2, obtained experimentally during the reactor testing.

Using the computed basis functions, the pressure at the observation hole can be calculated from the experimentally measured inlet and outlet pressures.

The gas flux through the observation window is related to the local pressure and gas velocity by Eq. (7). Since the gas expands into the low-pressure SEM chamber, the gas velocity cannot exceed the local speed of sound, providing a physically meaningful upper bound for the leakage flux.

Figure S7 presents the calculated ratio between the pressure at the observation window and the inlet pressure for six experimental conditions investigated in this work as a function of the assumed outlet velocity.

For gas velocities up to approximately 500 m s^{-1}, corresponding to the physically relevant operating range, the pressure at the observation window differs by less than 25% from the inlet pressure under all investigated experimental conditions.

These simulations indicate that the catalyst region observed by SEM experiences a pressure that remains close to the pressure imposed inside the reactor, thereby supporting the interpretation that the imaged catalyst is representative of the reaction environment within the packed bed.

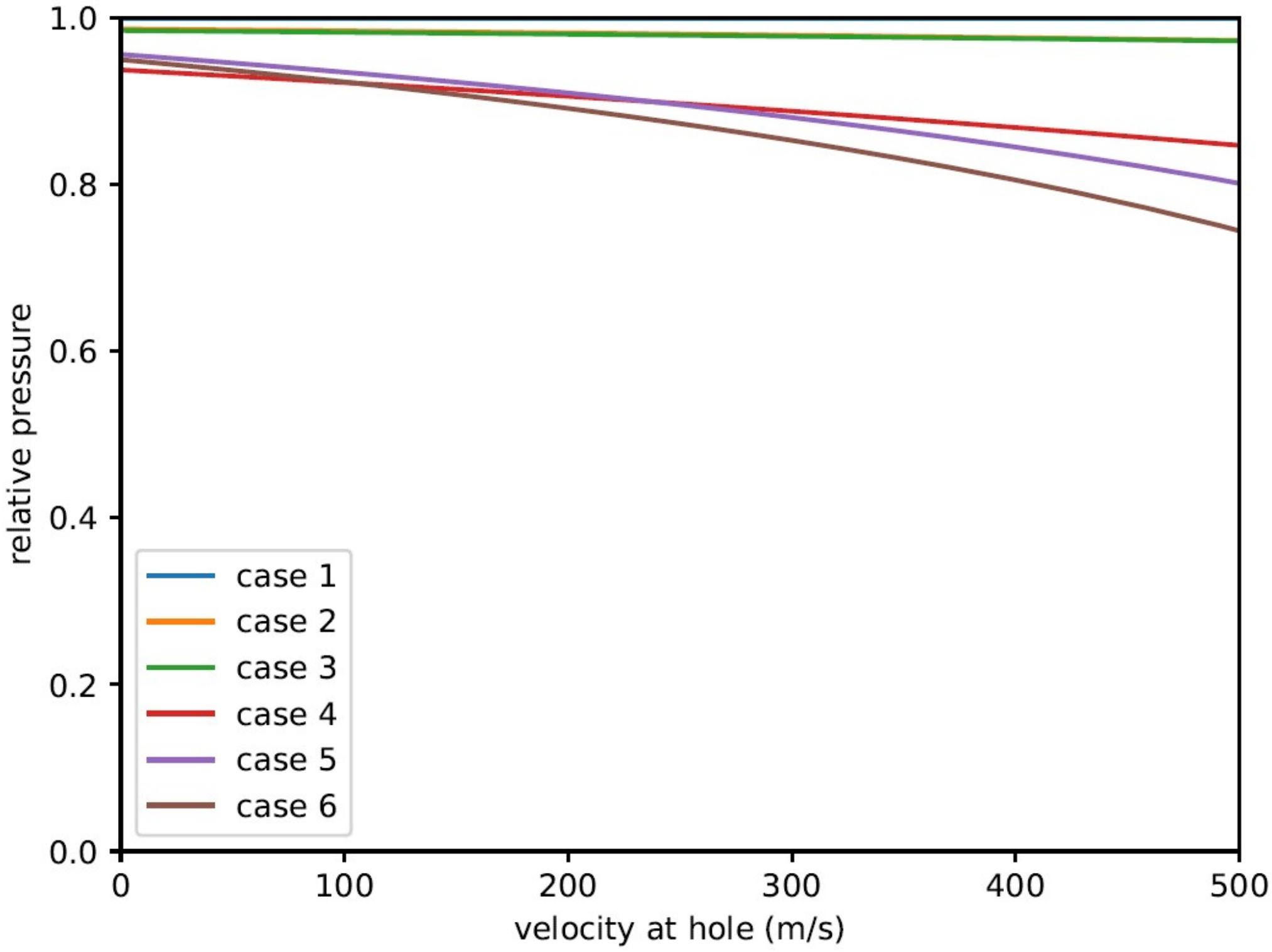

Fig. S7. A profile of the relative pressure value P_{hole}/P_{inlet} as a function of the leakage gas velocity at the viewing hole for the cases considered in Tab. S2. The Results support a pressure drop at the viewing hole of maximum 25% compared to the inlet pressure.

Table S2 also exemplifies estimated residence times in the PPR. The PPR contains a packed-bed geometry with a catalyst bed length of approximately 6 mm and an internal reactor diameter of 4 mm, corresponding to a geometric volume of approximately 75 µL. Considering a typical packed-bed porosity of 40%, the gas-accessible volume is estimated to be approximately 30 µL.

Using this value, an estimate of the residence time is

$$\tau = \frac{V_{void}}{Flow_{react}} \qquad (8)$$

where

$$Flow_{react} = Flow_N \frac{T_{react}}{T_N} \frac{P_N}{P_{react}} \qquad (9)$$

and $Flow_{react}$ is the volumetric gas flow rate corrected to the reaction temperature and pressure with respect to the normal conditions of the mass flow controllers at the feeding system. The exact residence time is more difficult to determine because it depends on the detailed catalyst packing, particle size distribution, local porosity, tortuosity of the bed and gas leakage through the viewing hole. A full description at this level of detail is out of our current scope.

According to the above relations, for constant temperature and inlet flow rate, the residence time scales approximately linearly with the reactor pressure. For the PPR2 experiments, where the temperature (250 °C) and inlet flow $Flow_N$ (9 Nml min^{-1}) were kept constant while the reactor pressure was varied, the estimated residence time increased from approximately 2.1 ms at 20 mbar to 4.9 ms at 43 mbar.

At the highest pressure reached in our current setup (120 mbar), a larger inlet flow of 30 Nml min^{-1} was required to sustain stable reactor operation. Under these conditions, the estimated residence time is

approximately 3.8 ms. Therefore, in the present configuration of PPR2, any increase in pressure is largely compensated by the higher gas flow, resulting in only a limited variation of the residence time.

For comparison, under otherwise identical temperature and inlet flow conditions (30 Nml min^{-1}) at atmospheric pressure and no viewing hole, i.e., in a conventional reactor, the estimated residence time would be approximately 31 ms. Consequently, operation at higher pressures and/or with deeper catalyst beds would be required to reproduce residence times comparable to those of conventional laboratory fixed-bed reactors.

For the original PPR, the estimated residence time was considerably shorter because of the much lower operating pressure. Moreover, at the sub-mbar pressures employed in PPR1, gas transport approaches the transitional (Knudsen) flow regime, where conventional residence-time estimates based solely on volumetric flow become less meaningful due to the diffusion interactions that start dominating the mass transport.

References

[1] M. K. Das, P. P. Mukherjee, and K. Muralidhar, Modeling transport phenomena in porous media with applications, Vol. 241 (Springer, 2018).

[2] S. Matera, C. Merdon, and D. Runge, Reduced basis approach for convection-diffusion equations with non-linear boundary reaction conditions, in Finite Volumes for Complex Applications X—Volume 1, Elliptic and Parabolic Problems, edited by E. Franck, J. Fuhrmann, V. Michel-Dansac, and L. Navoret (Springer Nature Switzerland, Cham, 2023) pp. 335–343.

[3] M. U. Qureshi, S. Matera, D. Runge, C. Merdon, J. Fuhrmann, J.-U. Repke, and G. Brösigke, Reduced order cfd modeling approach based on the asymptotic expansion—an application for heterogeneous catalytic systems, Chemical Engineering Journal 504, 158684 (2025).

[4] J. O. Hirschfelder, C. F. Curtiss, and R. B. Bird, The molecular theory of gases and liquids (John Wiley & Sons, 1964).

[5] J. Lang, Adaptive Multilevel Solution of Nonlinear Parabolic PDE Systems, Lecture Notes in Computational Science and Engineering, Vol. 16 (Springer Berlin Heidelberg, Berlin, Heidelberg, 2001).

[6] B. Erdmann, J. Lang, and R. Roitzsch, KARDOS - User's Guide, Tech. Rep. 02-42 (ZIB, Takustr. 7, 14195 Berlin, 2002).
